# The DNA Damage Repair Function of Fission Yeast CK1 Involves Targeting Arp8, a Subunit of the INO80 Chromatin Remodeling Complex

**DOI:** 10.1080/10985549.2024.2408016

**Published:** 2024-10-10

**Authors:** Sierra N. Cullati, Kazutoshi Akizuki, Yufan Shan, Eric Zhang, Liping Ren, Rodrigo X. Guillen, Lesley A. Turner, Jun-Song Chen, Jose Navarrete-Perea, Zachary C. Elmore, Steven P. Gygi, Kathleen L. Gould

**Affiliations:** a Department of Cell and Developmental Biology, Vanderbilt University School of Medicine, Nashville, Tennessee, USA; b Department of Cell Biology, Harvard Medical School, Boston, Massachusetts, USA

**Keywords:** CK1, casein kinase 1, *Schizosaccharomyces pombe*, DNA repair, nonhomologous end joining, homologous recombination, INO80, Arp8, phosphoproteomics

## Abstract

The CK1 family are conserved serine/threonine kinases with numerous substrates and cellular functions. The fission yeast CK1 orthologues Hhp1 and Hhp2 were first characterized as regulators of DNA repair, but the mechanism(s) by which CK1 activity promotes DNA repair had not been investigated. Here, we found that deleting Hhp1 and Hhp2 or inhibiting CK1 catalytic activities in yeast or in human cells increased double-strand breaks (DSBs). The primary pathways to repair DSBs, homologous recombination and nonhomologous end joining, were both less efficient in cells lacking Hhp1 and Hhp2 activity. To understand how Hhp1 and Hhp2 promote DNA damage repair, we identified new substrates of these enzymes using quantitative phosphoproteomics. We confirmed that Arp8, a component of the INO80 chromatin remodeling complex, is a bona fide substrate of Hhp1 and Hhp2 important for DNA repair. Our data suggest that Hhp1 and Hhp2 facilitate DNA repair by phosphorylating multiple substrates, including Arp8.

## Introduction

DNA damage poses a constant threat to cells by introducing alterations to the genetic code, impeding DNA replication, and contributing to genomic instability. Uncontrolled DNA damage is detrimental to cell survival, and in the case of metazoans, can contribute to cancer progression and therapeutic resistance. The cellular DNA damage response (DDR) consists of signaling proteins that recognize DNA damage, stall the cell cycle by activating cell cycle checkpoints, recruit DNA repair machinery, and ultimately repair DNA damage prior to replication or cell division. In addition to external hazards such as UV light and genotoxic chemicals, cells frequently experience DNA damage during normal cellular processes such as replication, transcription, and meiosis. For example, replication forks that stall due to DNA adducts, secondary structures, low nucleotide concentrations, or protein complexes that block their progression can lead to DNA damage when they collapse, including DNA double-stranded breaks (DSBs).^1–5^ DSBs are a particularly cytotoxic type of DNA damage, capable of not only obstructing DNA replication and gene expression, but also causing the loss of genetic information or instigating gross chromosomal rearrangements.^6^ The two primary DSB repair pathways are homologous recombination (HR), which requires a second copy of DNA for homology-directed repair, and nonhomologous end joining (NHEJ), which can directly ligate broken DNA ends, in addition to alternative repair pathways such as microhomology-mediated end-joining and single-strand annealing.

CK1 enzymes comprise a ubiquitous, conserved family of protein kinases with roles in many cellular processes, including the cell cycle, endocytosis, circadian rhythms, and stress responses.^7^ The mammalian CK1 family has seven isoforms (α1, α2, γ1, γ2, γ3, δ, ε) that are present to some degree in nearly every tissue type and cellular compartment, and they have hundreds of substrates, including some involved in the DDR such as p53, Mdm2, and claspin.^8–15^ In *Schizosaccharomyces pombe*, Hhp1 and Hhp2 are orthologues of mammalian CK1δ and CK1ε, as well as *Saccharomyces cerevisiae* Hrr25.^16^ Interestingly, *HRR25*, *hhp1*, and *hhp2* were all identified based on their sensitivities to methyl methanesulfonate (MMS) and γ-radiation.^17^^,^^18^ Cells missing *hhp1* and/or *hhp2* genes are also sensitive to a multitude of other DNA damaging agents including hydroxyurea (HU), ethyl methanesulfonate, 4-nitroquinoline N-oxide, bleomycin, UV, and ionizing radiation.^19^ In accord with their sensitivity to this wide array of specific and general DNA-damaging agents, cells lacking these enzymes are defective in repairing DSBs.^17^^,^^20^^,^^21^ Specifically, *hhp1Δ* and *hhp1Δ hhp2Δ* cells showed little ability to repair broken DNA caused by MMS or ionizing radiation as monitored by pulse-field gel electrophoresis.^17^ *hhp1* was also identified as a high copy suppressor of the *cdc24-M38* mutant.^22^ Cdc24 is required to complete DNA replication, interacts with PCNA, replication factor C, and Dna2, and is essential for repair of DSBs.^22–25^ Finally, Hhp1 was implicated in the response to broken replication forks caused by camptothecin (CPT) treatment.^26^ Subsequently, Hhp1 and Hhp2 were found to be involved in additional cellular processes including endocytosis, meiotic recombination, and delaying cytokinesis when spindle assembly is blocked.^27–32^

In this study, we focused on the previously deduced role of CK1 enzymes in DNA damage repair. We demonstrate that *S. pombe* and human cells lacking CK1 catalytic activity exhibit a significant increase in markers of DNA damage and a delay in entry into mitosis, and that eliminating a branch of the DNA damage checkpoint in yeast partially reduces the delay in mitotic entry that *hhp1Δ* and *hhp1Δ hhp2Δ* cells exhibit. Consistent with previous studies demonstrating a delay in DSB repair,^17^ we found that inhibiting analogue-sensitive mutants of Hhp1 and Hhp2 in *S. pombe* significantly impaired both HR and NHEJ, suggesting that these kinases promote DSB repair, among other possible roles in the DNA damage response. We employed quantitative phosphoproteomics to identify Hhp1 and Hhp2 substrates contributing to DNA damage repair and validated one new substrate of these enzymes, Arp8. Arp8 is a subunit of the INO80 chromatin remodeling complex, which evicts and slides nucleosomes around DSBs to promote end resection, an early step in DSB repair.^33–36^ Because DNA repair pathways and CK1 enzymes are conserved between *S. pombe* and other eukaryotes, we predict that our findings will yield insight into how CK1 promotes DNA repair in human cells.

## Results

### Loss of Hhp1 and Hhp2 kinase activity triggers the DNA damage checkpoint

*hhp1Δ* and *hhp1Δ hhp2Δ* strains are known to exhibit abnormally slow growth compared to wild-type.^17^^,^^37^^,^^38^ In addition to slow growth in liquid or solid media,^19^^,^^37^ *hhp1Δ* and *hhp1Δ hhp2Δ* have increased and unusually variable cell lengths at division (Supplementary material, Figure S1A and B).^17^^,^^26^ This is indicative of a G2 delay, and the G2 delay of *hhp1Δ* cells has been shown to be exacerbated when cells are treated with CPT.^26^ Deletion of *hhp2* alone does not result in these phenotypes, likely due to compensation by Hhp1, which is more abundant.^17^^,^^37^^,^^38^

*S. pombe* are rod-shaped yeast that grow from their cell tips during interphase, cease growth during mitosis, and divide medially at a set cell size; in this way, growth is coupled to the cell cycle, and cell cycle stage is closely correlated to cell length.^39^ When DNA damage occurs, *S. pombe* cells delay entry into mitosis via activation of checkpoints that inhibit Cdk1 activation, but growth continues during the delay, resulting in cells that divide at a longer size than usual.^40–42^ That *hhp1Δ* and *hhp1Δ hhp2Δ* cells have an increased length at septation suggests that they have activated one or more checkpoints to delay mitotic entry, possibly because of the presence of DNA damage in these strains.^17^^,^^37^ To test whether the DNA damage in the CK1 null strains activated a major DNA damage checkpoint leading to increased cell length, we deleted the master checkpoint kinase *rad3* in combination with *hhp1Δ*, *hhp2Δ*, or *hhp1Δ hhp2Δ*. Deleting *rad3* reduced the length of *hhp1Δ* and *hhp1Δ hhp2Δ* cells from an average of 18.3 ± 3.7 µm to 16.2 ± 3.6 µm and 23.2 ± 5.3 µm to 20.4 ± 5.9 µm, respectively (Supplementary material, Figure S1A and B). It also further impaired their growth (Supplementary material, Figure S1C), suggesting that the DNA damage checkpoint was involved in delaying entry into mitosis. However, the deletion of *rad3* did not return them to normal length nor lead to lethality (Supplementary material, Figure S1A to C). Rad3 operates upstream of both branches of the DNA damage checkpoint, activating Chk1 during the G2 DNA damage checkpoint and Cds1 during the intra-S phase replication checkpoint.^40^^,^^41^^,^^43^ We found that deletion of either *chk1* or *cds1* alone also reduced the length at septation of both *hhp1Δ* and *hhp1Δ hhp2Δ* cells, with *chk1Δ* having the largest effect (Supplementary material, Figure S1D). These data suggest that *hhp1Δ* and *hhp1Δ hhp2Δ* cells delay Cdk1 activation and entry into mitosis through both arms of the DDR as well as through an additional mechanism(s). There appear to be no significant defects in the ability of *hhp1Δ*, *hhp2Δ*, and *hhp1Δ hhp2Δ* cells to progress through mitosis or cell division as measured by the percentage of septated or binucleate cells in the populations (Supplementary material, Figure S1E and F) and at present, we do not know the identity of the additional mechanism(s) altering cell cycle progression outside of the DDR. Thus, we have focused here on better understanding the underlying defect in DNA damage repair in *hhp1Δ* and *hhp1Δ hhp2Δ* cells.

**Figure 1.**
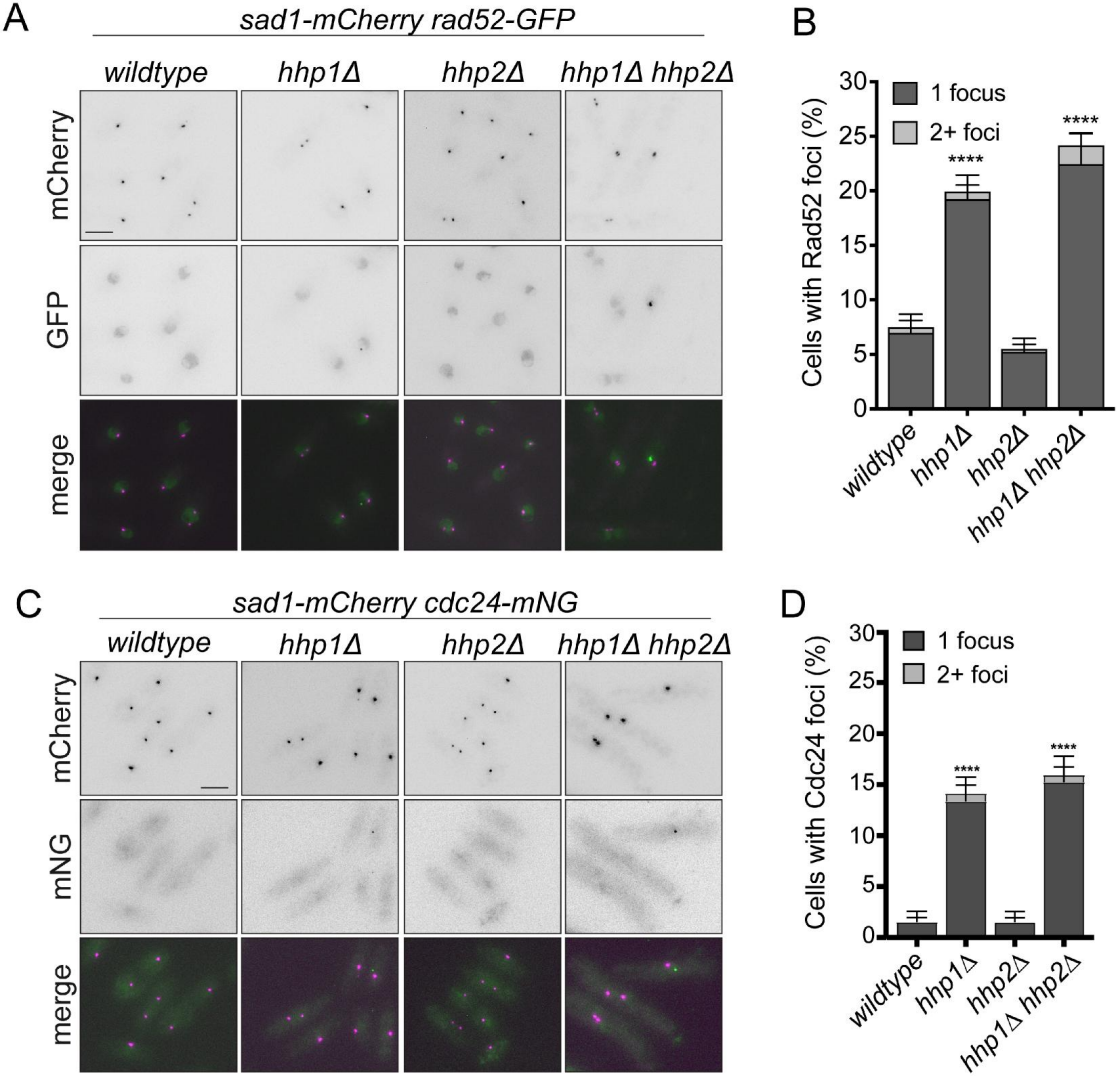
Deletion of *hhp1* alone or in combination with *hhp2* activates *rad3*-dependent DNA damage checkpoints. (A and C) Live-cell images of the indicated strains. Scale bars, 5 µm. (B and D) Rad52-GFP (B) and Cdc24-mNG (D) foci in > 600 cells for each strain were counted over two to four biological replicates. Error bars indicate ±95%CI. ****, *P* < 0.001 by chi-square.

Consistent with the previous reports of defects in DNA damage repair and the sensitivity of *hhp1Δ, hhp2Δ,* and *hhp1Δ hhp2Δ* cells to a broad spectrum of exogenous DNA damaging sources, including agents that modify bases (e.g., MMS), obstruct progression of the replication fork (e.g., HU), and induce DNA breaks (e.g., CPT),^17^^,^^20^^,^^21^ we observed that even without exposure to external DNA damaging agents, *hhp1Δ* and *hhp1Δ hhp2Δ* cells exhibited elevated levels of DNA damage, marked by the presence of Rad52-GFP foci (Figure 1A and B). The HR protein Rad52 localizes diffusely in the nucleus under normal conditions, while in response to various forms of DNA damage, including DSBs, nicks, and replication intermediates containing RPA-coated ssDNA, Rad52-GFP forms bright repair foci.^3^^,^^44–50^ Rad52-GFP protein levels were however unchanged in all strains examined (Supplementary material, Figure S1G). *hhp1Δ* and *hhp1Δ hhp2Δ* cells also had increased numbers of Cdc24 foci, another marker of DNA damage^25^^,^^51^ (Figure 1C and D). The spindle pole body (SPB) marker Sad1-mCherry was included to allow tracking of cell cycle progression. DNA damage foci were detected in only 8–13% of mitotic cells, defined as those cells with two SPBs, in all four strains with no significant differences among strains.

**Figure 2.**
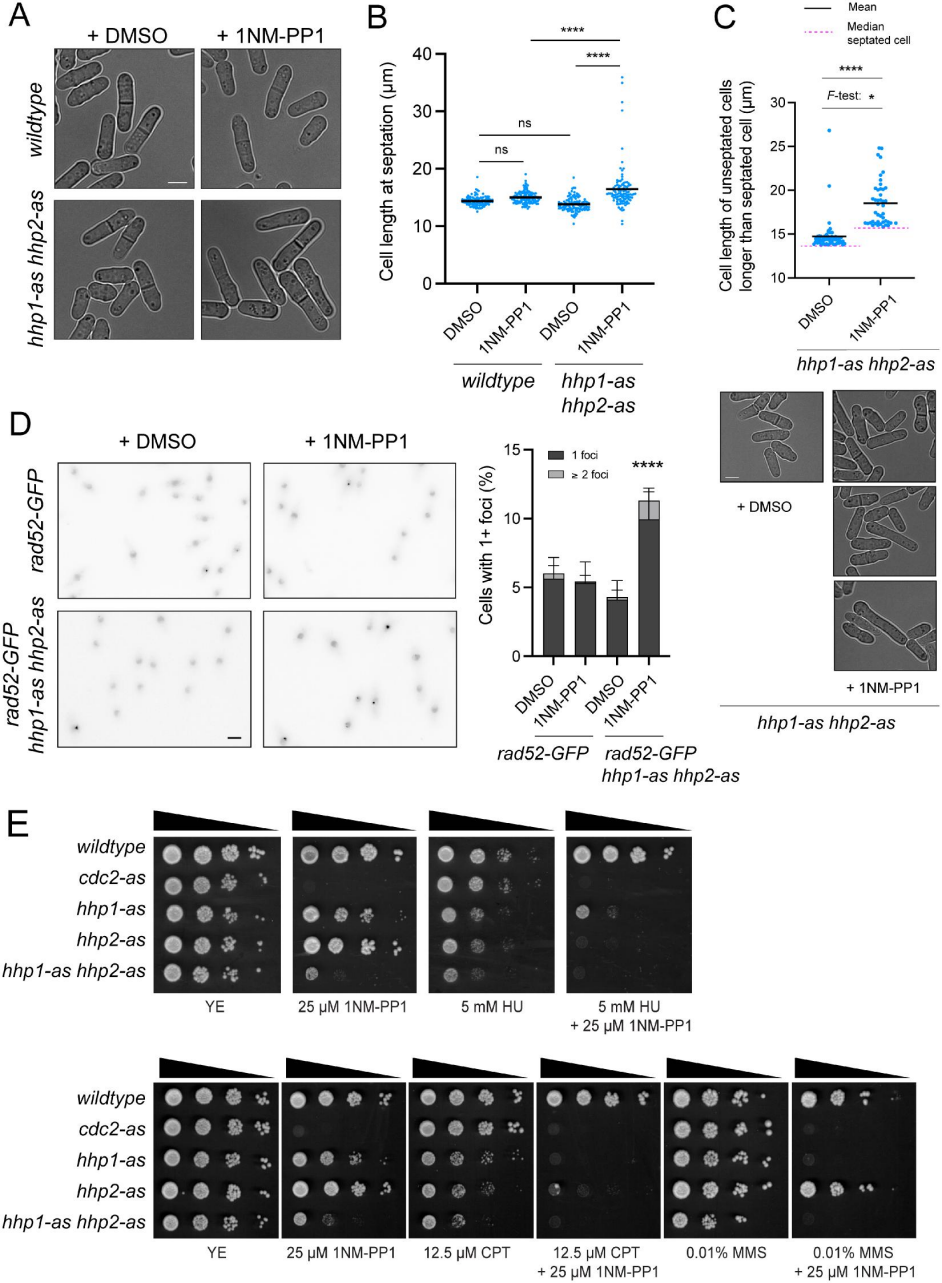
Loss of Hhp1 and Hhp2 activity triggers the DNA damage checkpoint. (A) DIC live-cell images of the indicated strains at 5 h after treatment with DMSO or 25 μM 1NM-PP1. Scale bar: 5 μm. (B) Length at septation was quantified for cells imaged as in A, *n* ≥ 100 cells. Bars represent means. ****, *P* < 0.0001 by one-way ANOVA; ns, not significant. (C) Length of cells without septum that are longer than the median length of the cells at septation from B. *n* ≥ 500 cells without septum were quantified and longer cells than the median are represented. Black solid bars represent means and magenta broken lines represent the median of septated cells from each treatment. *P < 0.05 by *F*-test; ****P < 0.0001 by Welch’s *t* test. Representative images are shown below. (D) Fluorescence microscopy images of *rad52-GFP* and *hhp1-as hhp2-as rad52-GFP* cells 5 h after treatment with DMSO or 25 μM 1NM-PP1. Rad52-GFP images are max projections. Scale bar: 5 μm. Rad52-GFP foci in > 1000 cells for each strain were counted over two biological replicates and are represented in the right graph. Error bars indicate ±95%CI. ****P < 0.001 by chi-square. (E) Serial 10-fold dilutions of the indicated strains were spotted on YE with or without 1NM-PP1 and hydroxyurea (HU), camptothecin (CPT), or methyl methanesulfonate (MMS), and incubated at 32 °C. *cdc2-as* was used as a positive control for inhibitor effectiveness; inhibition of Cdc2 is lethal.

Because of the severely compromised growth rates of the *hhp1Δ* and *hhp1Δ hhp2Δ* strains described above, we turned to using the *hhp1-M84G* and *hhp2-M85G* alleles that can be specifically inhibited by the ATP analogue 1NM-PP1 (denoted *hhp1-as* and *hhp2-as*).^27^^,^^52^^,^^53^ *hhp1-as hhp2-as* cells did not show the growth defects of the double null mutant, growing normally at a range of temperatures (Supplementary material, Figure S2A). However, when *hhp1-as hhp2-as* cells were treated with 25 µM 1NM-PP1 they elongated and exhibited an unusually variable length at division, like *hhp1Δ* and *hhp1Δ hhp2Δ* cells (Figure 2A and B and Supplementary material, Figure S1A and B). Because cells with DNA damage elongate and do not enter mitosis, the change in cell length could be underestimated. Therefore, we also quantified the length of unseptated cells and compared the distribution of the cells longer than the median length of the septated cells. In DMSO treated control cells, the lengths of *hhp1-as hhp2-as* cells were similar to those of the septated cells, as expected (Figure 2C). However, 1NM-PP1 treated, unseptated *hhp1-as hhp2-as* cells were more elongated with greater variance, suggesting that more cells continued growing before mitotic entry (Figure 2C). These cells also showed an increased number of Rad52-GFP foci compared to wild-type (Figure 2D). In accord, *hhp1-as* and especially *hhp1-as hhp2-as* cells grew poorly on agar plates containing 25 µM 1NM-PP1 (Figure 2E). Like the null mutants,^17^^,^^19–21^ the ATP analogue-sensitive strains grown on 25 µM 1NM-PP1-containing agar plates were sensitive to the DNA damaging agents HU, CPT, and MMS (Figure 2E). Similarly, kinase-dead *hhp1* and *hhp2* mutants^38^ were also sensitive to these agents (Supplementary material, Figure S2B).

### CK1 kinase activity promotes DSB repair in both yeast and human cells

**Figure 3.**
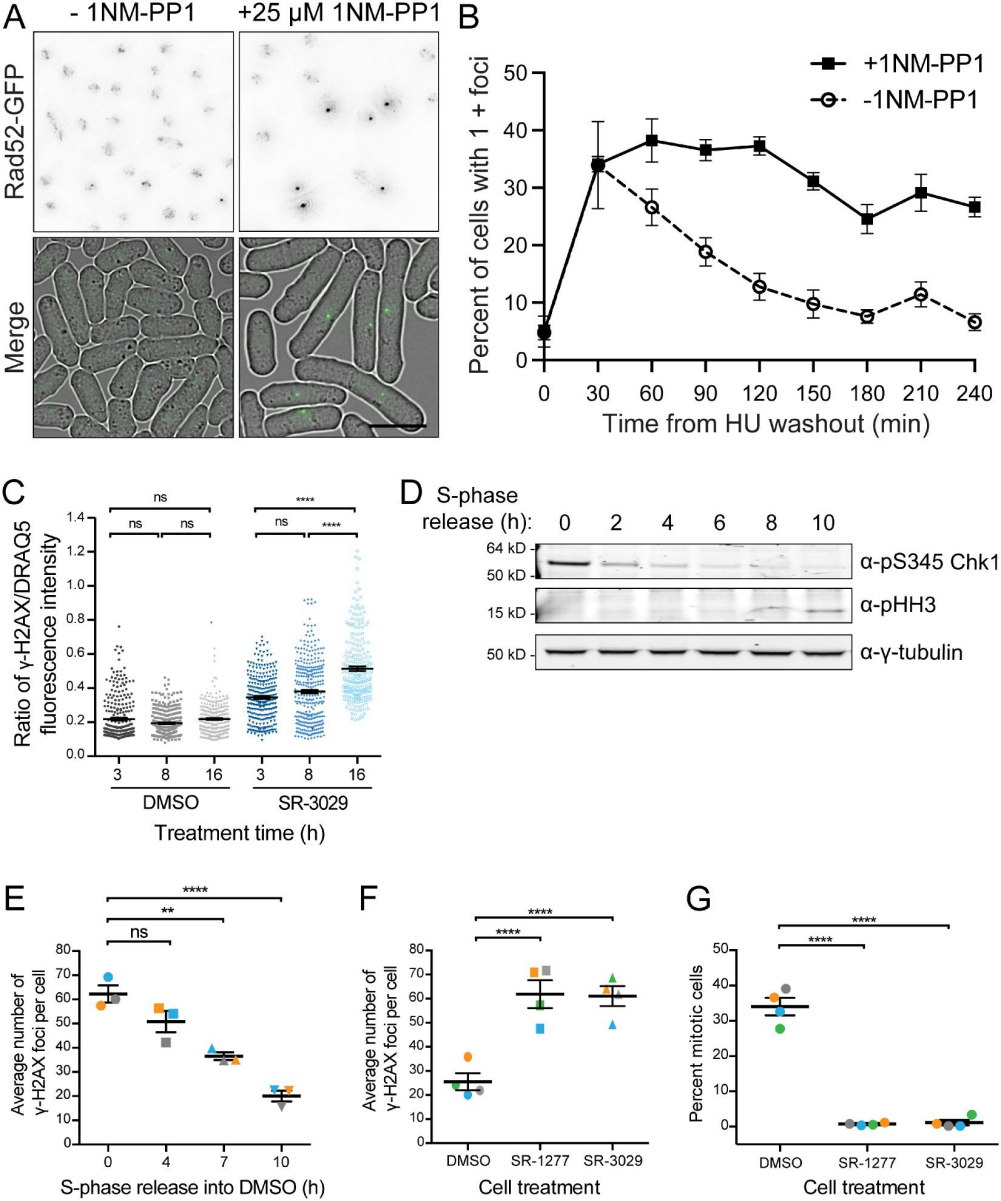
CK1 kinase activity promotes DSB repair in both yeast and human cells. (A and B) *hhp1-as hhp2-as rad52-GFP* cells were synchronized in 12 mM HU for 3 h then released into YE with or without 25 μM 1NM-PP1. Samples were fixed and imaged at the indicated timepoints. (A) Fluorescence microscopy and DIC images of the indicated strains after 240 min. Rad52-GFP images are max projections. DIC images are single medial slices. Scale bar: 10 μm. (B) Rad52-GFP foci were counted at the indicated timepoints using sum projections of cells imaged as in A. Data are means ± SEM from three replicates of *n* ≥ 150 each. (C) Asynchronous HeLa cells were treated with DMSO or 0.5 µM SR-3029 (CK1δ and CK1ε inhibitor) for the indicated times. Cells were fixed and stained for γH2AX as a marker of DSBs and DRAQ5 fluorescent probe for total DNA content. (D–G) HeLa cells were synchronized in S-phase using a thymidine-aphidicolin block. (D) Western blot of whole cell lysates with the indicated antibodies after release from S-phase arrest into DMSO. (E) Average number of γH2AX foci per cell after release from an S-phase arrest into DMSO. (F) Average number of γH2AX foci per cell 10 h after release from an S-phase arrest into DMSO, 0.5 µM SR-1227, or 0.5 µM SR-3029. (G) Mitotic index 10 h after release from an S-phase arrest into DMSO, 0.5 µM SR-1227, or 0.5 µM SR-3029.

To test whether the ATP analogue-sensitive mutants of Hhp1 and/or Hhp2 were defective in DNA damage repair like the null mutants,^17^ we used HU to both synchronize cells in S-phase and to induce replication stress, which indirectly leads to DNA damage. HU-treated *hhp1-as hhp2-as rad52-GFP* cells were then released into media without HU but containing 25 µM 1NM-PP1 or DMSO as a control, and cells were imaged over time to measure the number of Rad52-GFP foci (Figure 3A and B). Rad52-GFP rapidly localized to foci following release from HU, and cells with or without 1NM-PP1 treatment exhibited the same number of foci (Figure 3B), indicating that cells experience the same amount of DNA damage with and without Hhp1 and Hhp2 activity. However, while Rad52-GFP foci largely resolved within ∼2.5 h in uninhibited cells, they were maintained for more than 4 h in the presence of inhibitor (Figure 3A and B). Immediately after HU washout, the septation index was 2.6 ± 1.3% for untreated cells and 3.0 ± 2.2% for cells treated with inhibitor. During recovery from the HU arrest, neither population of cells entered mitosis; after 4 h, the septation index was 6.0 ± 1.4% and 6.5 ± 1.6% for untreated and treated cells, respectively, which is consistent with previous studies.^27^^,^^38^^,^^52^^,^^54^ These data indicate that cells lacking Hhp1 and Hhp2 kinase activity can still recognize DNA damage and initiate a cell cycle delay, but they are unable to complete DNA repair with normal kinetics during G2.

Because CK1 enzymes and the processes of DNA repair are conserved from yeast to human, we asked whether a similar DNA repair defect occurred when CK1δ and CK1ε, the human homologues of Hhp1 and Hhp2, were inhibited. γH2AX nuclear foci are a marker of DSBs in mammalian cells.^55^ We treated HeLa cells with SR-3029, a small molecule inhibitor of CK1δ and CK1ε,^56^ and observed accumulation of γH2AX foci over time, even without any treatment to induce DNA damage (Figure 3C). This resembled our results in *hhp1Δ* and *hhp1Δ hhp2Δ* fission yeast (Figure 1C and D) and a previous report in *CSNK1D^-/-^* MEFs.^57^ Next we synchronized cells in S-phase and induced replication stress using a thymidine-aphidicolin block and release. As expected, cells initially activated the DDR (Figure 3D), as evidenced by activated Chk1,^58^ due to a high number of DSBs (Figure 3E). Over time, DSBs were repaired (Figure 3E), satisfying the checkpoint, and by 10 h post-release cells were entering mitosis (Figure 3D), as evidenced by phosphohistone H3.^59^ However, when cells were released from the thymidine-aphidicolin block into CK1δ and CK1ε inhibitors SR-3029 and SR-1227,^56^ DSBs persisted for more than 10 h (Figure 3F), and cells failed to enter mitosis (Figure 3G). We conclude that like their yeast counterparts, CK1δ and CK1ε activities contribute to DNA damage repair.

### Hhp1 and Hhp2 phosphorylate multiple substrates to promote HR and NHEJ

**Figure 4.**
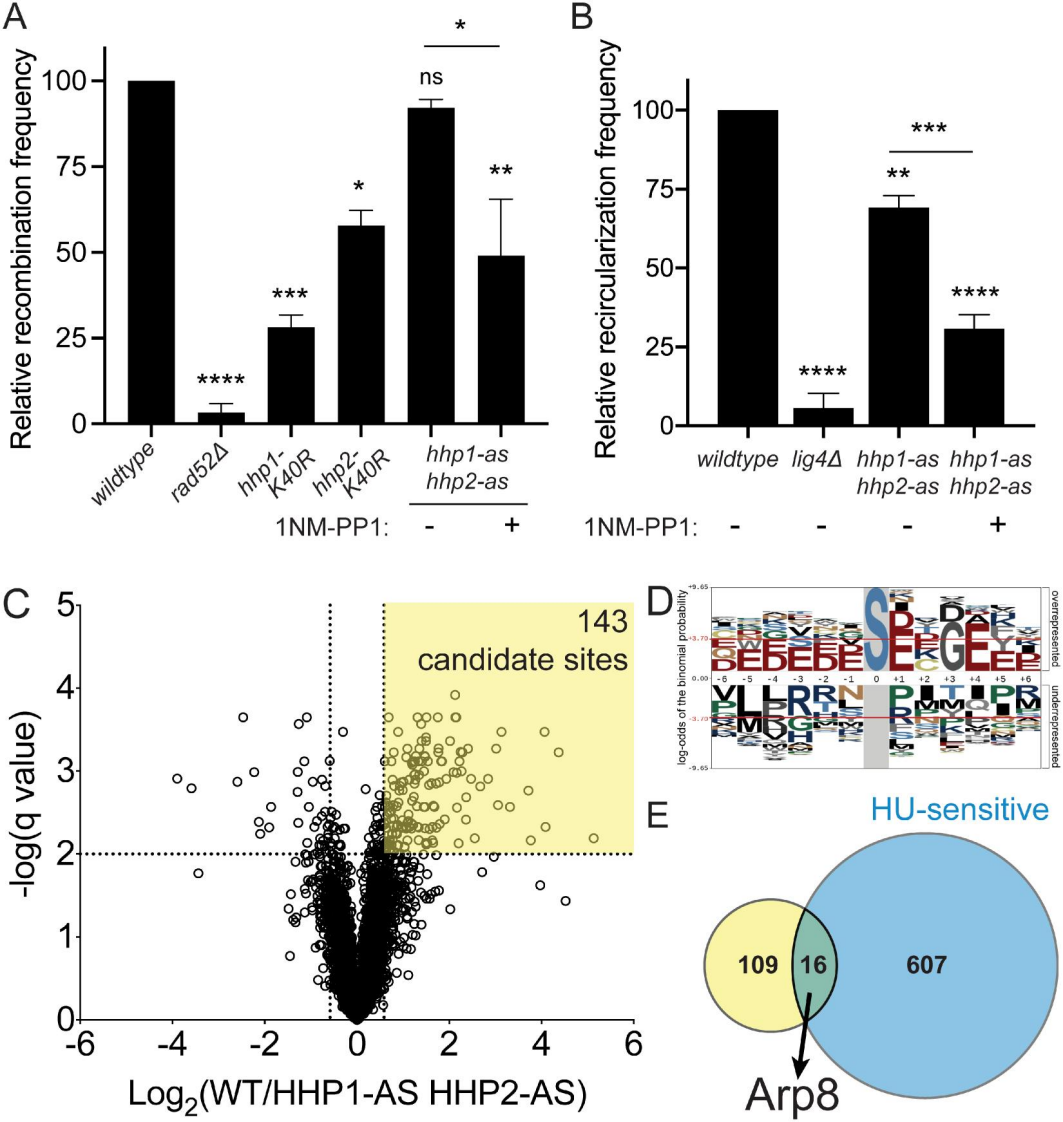
Hhp1 and Hhp2 phosphorylate multiple substrates to promote DSB repair through HR and NHEJ. (A) Analysis of spontaneous HR function using the RDUX200(+) reporter. Data presented as recombination frequency (ratio of colonies grown on selective media to rich media), normalized to wild-type. (B) Analysis of NHEJ function using a plasmid recircularization assay. Data presented as recircularization efficiency (ratio of colonies grown from cells transformed with linearized DNA to circular DNA on selective media), normalized to wild-type. *P < 0.05; **P < 0.01; ***P < 0.001; ****P < 0.0001 by one-way ANOVA. (C) Volcano plot comparing statistically significant differences in phosphorylation sites between *hhp1-as hhp1-as* relative to wild-type quantified by mass spectrometry-based phosphoproteomics. Dashed lines indicate a Benjamini–Hochberg-corrected *q* value < 0.01 and a fold change beyond ± 1.5. (D) Linear substrate motif analysis of phosphorylation sites with *q* < 0.01. (E) Overlap between candidate substrates identified by phosphoproteomics (yellow) and HU-sensitive alleles annotated in PomBase (blue). See also Tables S1–S3 (Supplementary material).

To understand how CK1 enzymes facilitate DNA damage repair, we focused on the function of Hhp1 and Hhp2. First, we sought to quantify the efficiencies of HR and NHEJ, the two major DSB repair pathways, in the presence and absence of Hhp1 and Hhp2 activity. We used the RDUX200(+) reporter to measure rates of spontaneous HR.^60^ This reporter gene consists of the *ura4* sequence interrupted by a kanamycin-resistance gene that is flanked by 200-base pair *ura4* repeats. Homologous copies of these *ura4* sequences can recombine to delete the *kanMX6* gene, yielding an intact *ura4^+^* gene. Colony growth on media lacking uracil is then used to quantify the rate of recombination. Deletion of *rad52*, which is required for HR,^45^^,^^47^ prevents nearly all recombination, and inhibiting *hhp1-as hhp2-as* cells significantly reduced the frequency of HR to about half that of uninhibited *hhp1-as hhp2-as* and wild-type cells (Figure 4A). We also assayed cells completing lacking either Hhp1 or Hhp2 function individually (Hhp1-K40R and Hhp2-K41R are inactive kinases^38^) and found that both strains were deficient in HR (Figure 4A).

To quantify NHEJ function, we used a plasmid recircularization assay, in which a linearized plasmid carrying a *ura4* expression cassette is transformed into cells.^61^^,^^62^ In order to express *ura4* and survive on uracil-deficient media, cells must recircularize the plasmid using NHEJ. To correct for different transformation efficiencies across strains, cells are also transformed with the circular plasmid, and the linear to circular ratio is calculated for each to yield the frequency of NHEJ. Deletion of *lig4*, the ligase gene required for the final step of NHEJ,^63^ prevented recircularization (Figure 4B). *hhp1-as hhp2-as* cells, which are slightly hypomorphic even without inhibitor, moderately reduced NHEJ frequency, and treating these cells with 1NM-PP1 further reduced NHEJ to about 30% of wild-type levels (Figure 4B). Deletion of *hhp1* had a similar effect to inhibition of *hhp1-as hhp2-as* (Supplementary material, Figure S2C). These data indicate that both major DSB repair pathways are less efficient in *hhp1* and *hhp2* mutants.

Hhp1 and Hhp2 localize diffusely across the nucleus and cytoplasm with a concentration at SPBs,^38^ but they did not co-localize with Rad52-GFP foci, even when genotoxic stress was induced (Supplementary material, Figure S2D). This suggests that their repair function may occur distally from sites of damage and thus we took a global approach to identify Hhp1 and Hhp2 substrates impacting DNA damage repair efficiency. Specifically, we used the *hhp1-as hhp2-as* strain and 1NM-PP1 inhibition in combination with multiplexed quantitative phosphoproteomics (SL-TMT).^64^ To correct for any changes in protein abundance, we also analyzed total proteome samples (Supplementary material, Table S1). Each strain was analyzed in biological triplicate. We quantified a total of 4336 phosphorylation sites on 1598 proteins (Supplementary material, Table S2). Phosphopeptides corresponding to 143 phosphorylation sites on 125 proteins decreased significantly by more than 1.5-fold after inhibition of Hhp1 and Hhp2, representing candidate substrates (Figure 4C). These sites were enriched for the CK1 consensus motif,^65^^,^^66^ suggesting that they are likely to contain direct substrates (Figure 4D).

Because loss of Hhp1 and Hhp2 activity results in sensitivity to DNA damaging drugs and deficiency in DNA repair (Figures 2E and 3A and B), we reasoned that loss of Hhp1 and Hhp2 substrates would confer similar sensitivities if they were involved in DNA repair. Therefore, we cross-referenced the candidate substrates identified by phosphoproteomics with alleles that have been annotated as drug-sensitive in PomBase, the Global Core Biodata Resource for *S. pombe*.^67^ We began with HU because of the striking sensitivity of *hhp1Δ*, *hhp2 Δ*, and *hhp1Δ hhp2Δ* cells to HU^17^^,^^20^^,^^21^ and the dramatic delay in Rad52 foci resolution we observed in *hhp1-as hhp2-as* cells following HU treatment (Figure 3B). This resulted in 20 phosphorylation sites on 16 proteins (Figure 4E and Supplementary material, Table S3). Some of these proteins were poorly characterized and/or not conserved in higher eukaryotes, though several were conserved DNA binding proteins (e.g., ATP-dependent helicase Upf1, DNA damage response protein Mdb1, single-stranded DNA binding protein Tcg1, transcription factor Pap1) (Supplementary material, Table S3). We focused on the actin-related protein Arp8 for further investigation because it contained three phosphorylation sites that were strongly downregulated in *hhp1-as hhp2-as* cells and which all resembled the CK1 motif ((pS/pT)xxS or (D/E)xxS) (Supplementary material, Table S1), and *arp8Δ* cells are sensitive to additional DNA damaging agents, like *hhp1Δ* cells.^19^ In addition, previous literature established a role for Arp8 in DSB repair,^33–36^^,^^68–71^ its function could explain defects in both HR and NHEJ,^33^^,^^69^^,^^71^^,^^72^ and it is conserved from yeast to human (human orthologue *ACTR8*).

### Arp8, a subunit of the INO80 complex, is an Hhp1 and Hhp2 substrate that is important for DSB repair

Arp8 is a conserved subunit of the INO80 complex, an ATP-dependent nucleosome remodeler that regulates access to the genome during DNA replication, transcription, and DNA repair.^72^ Arp8 facilitates INO80 binding to DNA, including at DSBs.^33^^,^^34^^,^^36^^,^^68–71^^,^^73^ Furthermore, Arp8 promotes end resection at DSBs during both NHEJ^36^ and HR,^34^^,^^35^ Rad51 filament formation and eviction of H2AZ during HR,^35^ and the nucleosome sliding activity of INO80.^33^

**Figure 5.**
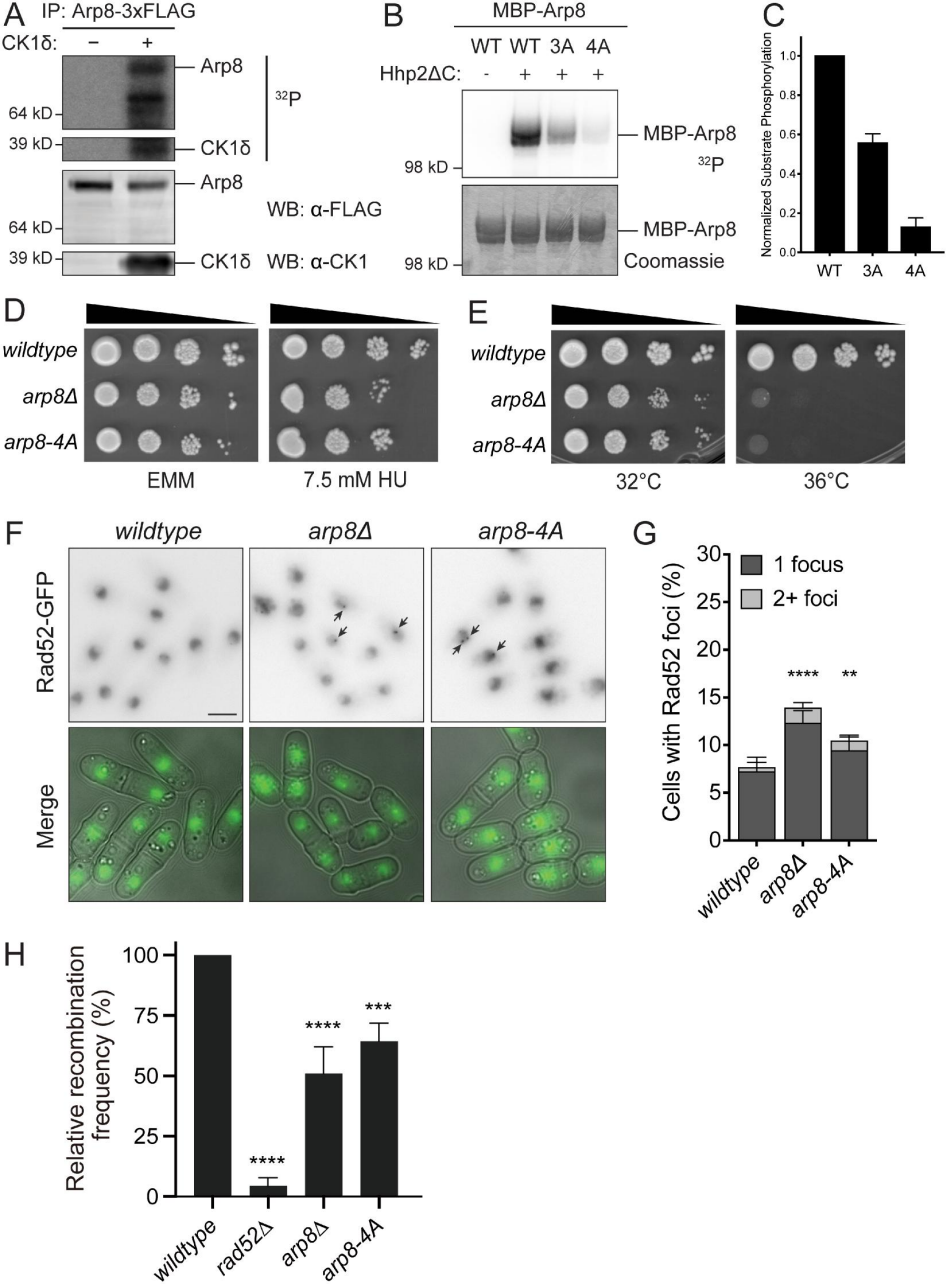
Arp8, a subunit of the INO80 complex, is a substrate of Hhp1 and Hhp2. (A) Arp8-3 × FLAG was immunoprecipitated from *S. pombe* then incubated with or without recombinant CK1δ at 30 °C for 1 h. Phosphorylated proteins were detected by autoradiography (^32^P) and protein levels by Western blot with the indicated antibodies. (B and C) Recombinant Arp8 mutants were incubated with recombinant Hhp2ΔC at 30 °C for 1 h. Phosphorylated proteins were detected by autoradiography (^32^P) and protein levels by Coomassie. (C) Bars represent means ± SD from four independent replicates. ^32^P signal was normalized to protein level for each lane. (D) Serial 10-fold dilutions of the indicated strains were spotted on EMM ± HU and incubated at 32 °C. (E) Serial 10-fold dilutions of the indicated strains were spotted on YE and incubated at the indicated temperatures. (F and G) *arp8Δ* and *arp8-4A* exhibit increased basal levels of Rad52-GFP foci compared to wild-type. (F) Live-cell images of the indicated strains. Merge represents DIC and GFP channels. Rad52-GFP images are sum projections. DIC images are single medial slices. Scale bar, 5 µm. (G) Rad52-GFP foci in > 1300 cells for each strain were counted over three biological replicates. Error bars indicate ±95%CI. ****P < 0.001; **P < 0.01 by chi-square. (H) Spontaneous HR recombination frequency of the indicated strains, normalized to wild-type. ***P < 0.001; ****P < 0.0001 by one-way ANOVA.

To confirm that Arp8 could be phosphorylated by CK1, we tagged *arp8* at the endogenous locus with 3 × FLAG, immunoprecipitated the protein from *S. pombe* cells, and incubated with recombinant CK1δ. In accord with our phosphoproteomics results that suggested CK1-mediated phosphorylation of Arp8 in vivo, this in vitro kinase assay also demonstrated phosphorylation of Arp8 (Figure 5A). Next we tested whether the three phosphorylation sites identified by phosphoproteomics (S62, S75, and S87) were indeed where Hhp1 and Hhp2 were phosphorylating Arp8. We expressed and purified recombinant Arp8-WT and Arp8-3A, which has S62, S75, and S87 mutated to alanine, and performed in vitro kinase assays using recombinant Hhp2ΔC, which has the C-terminus truncated to generate a highly active kinase.^74–77^ Mutating S62, S75, and S87 to alanine decreased Arp8 phosphorylation, indicating that Hhp2 phosphorylates these sites in vitro; however, Arp8-3A was still phosphorylated, suggesting the presence of additional sites that had not been detected in the phosphoproteomics screen (Figure 5B and C).

To identify the remaining site(s), we analyzed in vitro phosphorylated Arp8 and Arp8-3A by LC-MS^3^. This identified nine phosphorylated serines, including S62 and S87, and one additional serine S60 that had both high spectral counts and the CK1 motif. We expressed and purified recombinant Arp8-4A, which had S60 mutated to alanine along with S62, S75, and S87. When incubated with Hhp2ΔC in vitro, Arp8-3A reduced phosphorylation to ∼60% of the wild-type level and introducing S60A further reduced phosphorylation to ∼10% (Figure 5B and C). S60, along with S62 and S87, was also detected in previous phosphoproteomics experiments.^78^^,^^79^ These data strongly suggest that S60 is an additional Hhp1 and Hhp2 phosphorylation site, and that S60, S62, S75, and S87 are the primary sites on Arp8 that are targeted by Hhp1 and Hhp2.

To confirm that fission yeast Arp8 promotes DNA repair and to test whether phosphorylation by Hhp1 and Hhp2 is important for its DNA repair function, we grew *arp8Δ* and *arp8-4A* cells on media containing HU (Figure 5D). Consistent with previous literature, *arp8Δ* was slightly sensitive to HU and very sensitive to high temperature (Figure 5E).^80^^,^^81^ *arp8-4A* was also HU- and temperature-sensitive, suggesting that loss of Hhp1 and Hhp2 phosphorylation generated an *arp8* loss-of-function allele (Figure 5D and E). Furthermore, *arp8Δ* and *arp8-4A* had statistically significant increases in the number of cells with Rad52-GFP foci (Figure 5F and G). Approximately 15% of *arp8Δ* cells and ∼11% of *arp8-4A* cells had one or more foci, suggesting that loss of *arp8* function prevents cells from efficiently repairing DNA damage, and that phosphorylation by Hhp1 and Hhp2 is critical for this function. Next we measured the efficiency of HR in *arp8Δ* and *arp8-4A* strains. *arp8Δ* and *arp8-4A* were both deficient in HR, though their recombination frequencies were greater than with loss of CK1 activity, as would be expected if Arp8 were not the only CK1 target involved in DNA repair (Figure 5H). *arp8-4A* had a greater recombination frequency than *arp8Δ*, also as predicted by the difference between a hypomorphic and null mutant (Figure 5H). Consistent with our results, deletion of *arp8* in budding yeast produced a similar recombination defect.^35^ We found that *arp8Δ* and *arp8-4A* strains were very difficult to transform with exogenous DNA (transformation efficiencies of 1.8% and 10% of wild-type for *arp8Δ* and *arp8-4A*, respectively), precluding us from measuring plasmid recircularization by NHEJ as we had for *hhp1-as hhp2-as* and *hhp1Δ*; however, previous literature has shown that the budding yeast Arp8 plays a minor role in error-prone NHEJ following an induced DSB.^36^ Indeed, given the milder defects of *arp8Δ* and *arp8-4A* with respect to DNA damage sensitivity and repair function compared with *hhp1* and *hhp2* mutants, it is likely that Hhp1 and Hhp2 have additional substrates, potentially some that we identified in our phosphoproteomics screen, involved in protecting genome integrity.

## Discussion

In this study, we have expanded our understanding of Hhp1 and Hhp2’s function in fission yeast DNA damage repair. Our data demonstrate that Hhp1 and Hhp2 mutants inefficiently repair DNA damage, both with and without external sources of genotoxic stress. Cells lacking Hhp1 and Hhp2 activity are still capable of initiating the DDR, as evidenced by the recruitment of early repair proteins such as Rad52 and delaying the cell cycle. However, cells deficient in Hhp1 and Hhp2 activity are unable to complete repair in a timely manner. The molecular players in DNA repair are conserved from yeast to human, and we see similar accumulation of DSBs in γH2AX repair foci and cell cycle checkpoint activation in human cells treated with CK1δ and CK1ε inhibitors.

The repair defect in Hhp1 and Hhp2 mutants is pleiotropic, affecting at least two major repair pathways, HR and NHEJ. HR and NHEJ are co-regulated across the cell-cycle, as they compete for broken DNA substrates, and these pathways have been found to share a handful of early DDR proteins, e.g., Mre11, histone H2AX, and Rad3.^82–84^ However, the core proteins responsible for directly engaging repair of DSBs in each pathway are distinct, suggesting that Hhp1 and Hhp2 may have different substrates in HR and NHEJ. This is consistent with the sensitivity of cells lacking Hhp1 and Hhp2 activity to a wide variety of DNA damaging agents. In addition, our proteomics data identified multiple candidate substrates that are known to have roles in DNA repair (Figure 4E).

We focused on Arp8 because of its well-established role in promoting the function of INO80 during DSB repair by both NHEJ and HR pathways, and because the change in Arp8 phosphorylation upon inhibition of Hhp1 and Hhp2 was pronounced. The four serines phosphorylated by Hhp1 and Hhp2 all reside in the N-terminus of Arp8. The AlphaFold2 prediction of Arp8 structure^85^^,^^86^ shows that the N-terminus is poorly predicted and likely flexible; this could be due to or enhanced by different patterns of phosphorylation. In budding yeast, the Arp8 N-terminus was shown to bind to linker DNA between nucleosomes and regulate the nucleosome sliding function of INO80.^33^ Deleting the N-terminus of Arp8 resulted in HU-sensitivity similar to *arp8Δ*,^33^ demonstrating its essential role in DNA repair. In *S. pombe*, phosphorylation of this region of Arp8 could affect the assembly of the INO80 complex, its localization to DSBs, and/or its nucleosome remodeling activity. We predict that following DNA damage, Hhp1 and Hhp2 phosphorylate Arp8, which opens the chromatin surrounding DSBs through the nucleosome sliding and evicting activity of INO80, making breaks accessible to the repair machinery for HR and NHEJ.

Future studies will determine the mechanism by which Hhp1 and Hhp2 phosphorylation affects the function of Arp8 and INO80, and how this is regulated under different cellular conditions. Future work will also investigate additional substrates that CK1 targets in response to DNA damage. Given the conservation of DNA repair processes and CK1 function from yeast to human, and the similar phenotypes we observed between *S. pombe* and cultured human cells (Figure 3), these findings are likely to also apply to higher eukaryotes.

## Materials and Methods

### Yeast methods

*S. pombe* strains used in this study (Supplementary material, Table S4) were grown in yeast extract with supplements (YE) at 32 °C unless otherwise indicated. 3′ ORF tagging and gene knockout or disruption was achieved by lithium acetate transformation of PCR-amplified fragments containing regions of homology to the locus of interest.^87^ Genes were tagged at their 3′ end using pFA6 cassettes as previously described.^88^ Strains were constructed using standard *S. pombe* crossing and tetrad dissection techniques.^89^ The ATP analogue-sensitive mutants *hhp1-M84G:kanMX6* and *hhp2-M85G:natMX6* and the *arp8-4A* phosphorylation site mutant were generated in pIRT2 constructs using site-directed mutagenesis, and subsequently integrated over the null using a lithium-acetate protocol. *hhp1-M84G:hphMX6 hhp2-M85G:natMX6* was constructed by swapping the *kanMX6* marker for *hphMX6* by lithium acetate transformation. Inhibition of analogue-sensitive Hhp1 and Hhp2 was achieved by exposing cells to 25 μM 1NM-PP1 for 1 h or 5 h in liquid YE or by growing 2–4 d on YE agar containing 25 μM 1NM-PP1. For growth assays, three 10-fold serial dilutions were made in sterile water starting at 6 × 10^6^ cells/mL. Three microliters of each dilution was spotted on YE plates with the indicated concentrations of DNA damaging agents, and cells were grown at the indicated temperatures.

### Microscopy methods

Live-cell images of *S. pombe* used for length measurements were acquired with a spinning disk confocal microscope (Ultraview LCI; PerkinElmer, Waltham, MA) equipped with a 63 × 1.46 NA PlanApochromat oil immersion objective (Zeiss), an EM-CCD ImagEM X2 camera (Hamamatsu) and μManager software. Strains in the *hhp1-M84G hhp2-M85G* background were treated for 5 h with 25 μM 1NM-PP1 in YE; samples were taken before and after treatment for imaging. Length was measured from a single medial slice in Fiji^90^ by drawing line segments between each pole of the cell along the midpoint of the diameter to determine the total cell length at septation or no septation.

Representative live-cell images and fixed-cell fluorescence images of *S. pombe* and HeLa cells were acquired using a Personal DeltaVision (Leica Microsystems) that includes a microscope (IX71; Olympus), 60× NA 1.42 Plan Apochromat objective, fixed and live-cell filter wheels, a camera (CoolSNAP HQ2; Photometrics), and softWoRx imaging software (Leica Microsystems). For fixed-cell fluorescence imaging of Rad52-GFP foci in the *hhp1-M84G hhp2-M85G* background, strains were treated with 12 mM HU for 3 h and then washed into YE containing either 25 μM 1NM-PP1 or DMSO at 32 °C. Samples were taken every 30 min for 4 h and fixed with ice-cold 70% ethanol on ice for 15 min. Fixed cells were washed three times in phosphate-buffered saline (PBS) prior to imaging. Eleven z-sections spaced at 0.5 μm were acquired for each image.

Live-cell images of Rad52-GFP and Cdc24-mNG foci were acquired using a Zeiss Axio Observer inverted epifluorescence microscope which includes an AxioCam 503 mono camera with Zeiss Plan Apochromat 63× oil (1.46 NA) objective and captured and deconvolved using Zeiss ZEN 3.0 (Blue edition) software. The z-stack step size was 0.25 µm and a total of 19 Z-slices were acquired. Images shown were max projections. Foci were quantified as described previously in Fiji^90^ by applying a Fire heatmap to sum projections of nondeconvolved images.^2^ The percentage of cells containing at least one yellow/orange/white focus was counted for each cell population. More than 600 cells for each strain were imaged over two to four independent replicates and pooled.

### Human cell culture and synchronization

HeLa cells were cultured in Dulbeco’s modified eagle medium (DMEM) supplemented with 10% fetal bovine serum and 1% penicillin/streptomycin. Cells were synchronized using a sequential thymidine (2.5 mM; Sigma-Aldrich) and aphidicolin (5 μg/mL; Tocris Bioscience) block-and-release protocol. Stock solutions of SR-1227 and SR-3029 were prepared in DMSO; a corresponding volume of DMSO was used for all negative controls.

For microscopy, cells were seeded onto 25 mm coverslips that were contained in six-well plates. Cells were washed once with cold PBS and once with 100% cold methanol, followed by fixation with 100% cold methanol for 15 min at −20 °C. Cells were then washed three times with PBS + 0.1% Tween-20 at RT, followed by blocking with 2% normal goat serum in 0.1% Triton-X100 in PBS for 10 min. Cells were incubated with rabbit anti-γH2AX (Cell Signaling Technology, 2577S), then DRAQ5 (Thermo Fisher Scientific) or DAPI and secondary antibodies (Alexa Fluor Goat-anti Rabbit IgG (H + L), ThermoFisher; 1:500) for 45 min at RT. Coverslips were mounted on slides using Prolong Gold antifade mounting media.

For Western blotting, ∼40 μg whole cell lysate was incubated with antibodies against γ-tubulin (Sigma-Aldrich, GTU88), phospho-Chk1 (Cell Signaling Technology, 2348S), or phospho-Histone H3 (Sigma, H0412).

### Nonhomologous end joining (NHEJ) plasmid recircularization assay

pFY20^61^ was linearized by PstI (New England Biolabs) digest, and complete digestion was confirmed by agarose gel electrophoresis. Prior to transformation, *hhp1-M84G hhp2-M85G* cells were treated with either 25 μM 1NM-PP1 for 1 h at 32 °C or a DMSO vehicle control; all other strains were treated with DMSO for 1 h. Cells were transformed with linear, PstI-digested pFY20 or circular, uncut pFY20 via electroporation (Bio-Rad Gene Pulser). Transformants were subsequently grown on Edinburgh minimal media (EMM) lacking uracil at 32 °C for 4 d. Colonies were counted, and the linear/circular ratio (L/C) for each strain was calculated to represent the recircularization efficiency.

### Homologous recombination (HR) assay

The RDUX200(+) reporter system, consisting of an interruption of the endogenous *ura4* locus with a *kanMX6* insertion flanked by 200 bp tandem repeats, was used to measure rates of spontaneous recombination within different strain backgrounds.^60^ Strains were grown to log phase overnight in YE with G418 (100 μg/mL) to eliminate pre-existing recombinants. *hhp1-M84G hhp2-M85G* cells were either treated with 25 μM 1NM-PP1 for 1 h at 32 °C or a DMSO vehicle control; all other strains were treated with DMSO for 1 h. Cells were washed with sterile water, diluted, and plated on EMM lacking uracil and YE. Recombination frequency was determined by the ratio of colonies grown on uracil-free media to those grown on YE.

### Protein purification

*Escherichia coli* Rosetta2(DE3)pLysS cells were grown in terrific broth (TB) to an OD of 1.2. Protein production was induced by addition of 0.1 mM IPTG overnight at 17 °C. Cells were lysed in lysis buffer (20 mM Tris pH 7.4, 150 mM NaCl, 1 mM EDTA, 0.1% NP-40, 1 mM DTT, 1 mM PMSF, 1.3 mM benzamidine, protease inhibitor tablets; Roche) using 300 μg/mL lysozyme for 20 min followed by sonication. MBP-Arp8 proteins were purified on an MBPTrap HP column (Cytiva) in column buffer (20 mM Tris pH 7.4, 150 mM NaCl, 1 mM EDTA, 1 mM DTT, 1 mM PMSF, 1.3 mM benzamidine) and eluted with maltose (20 mM Tris pH 7.4, 150 mM NaCl, 1 mM EDTA, 1 mM DTT, 1 mM PMSF, 1.3 mM benzamidine, 10 mM maltose, 10% glycerol). Hhp1ΔC and Hhp2ΔC were purified as described previously.^52^

### Immunoprecipitation

*S. pombe* strains were grown in YE to log phase. Cell pellets were washed once in NP-40 buffer (10 mM NaPO4 pH = 7.0, 1% NP-40, 150 mM NaCl, 2 mM EDTA, 50 mM NaF, 4 μg/mL leupeptin, 100 mM Na_3_VO_4_, 1 mM PMSF, 1.3 mM benzamidine, protease inhibitor tablets [Roche], phosphatase inhibitor tablets; Roche) and lysed by bead disruption using a FastPrep cell homogenizer (MP Biomedicals). The homogenized cell/bead mixture was treated with 500 μL SDS lysis buffer (10 mM sodium phosphate, pH 7.0, 1% SDS, 1 mM DTT, 1 mM EDTA, 50 mM NaF, 100 μM sodium orthovanadate, 1 mM PMSF, 4 μg/mL leupeptin) and incubated at 95 °C for 2 min. Lysate was extracted with 800 μL NP-40 buffer and cleared by centrifugation at 17,000 × *g* for 15 min at 4 °C. Arp8-3xFLAG was immunoprecipitated using 2 μg FLAG-M2 (Sigma) at 4 °C for 1 h, followed by the addition of Protein A Sepharose beads (GE Healthcare) for 30 min. Proteins were eluted by boiling in SDS-PAGE sample buffer. Samples were separated by SDS-PAGE, transferred to Immobilon-P polyvinylidene fluoride membrane (Millipore) at 30 V for 1 h, and immunoblotted with mouse anti-FLAG (Sigma) and goat anti-mouse fluorescent antibody (Li-Cor Biosciences), and imaged on an Odyssey CLx (Li-Cor Biosciences).

### In vitro kinase assays

For in vitro kinase assays on immunoprecipitated proteins, beads were equilibrated in 1× PMP buffer (50 mM HEPES, 100 mM NaCl, 2 mM DTT, 0.01% Brij 35, pH 7.5; New England Biolabs). Kinase reactions were performed with 1 μL commercial CK1δ (New England Biolabs) in 1× PMP buffer supplemented with 100 μM unlabeled ATP, 0.5 μCi γ-[^32^P]-ATP, and 10 mM MgCl_2_ at 30 °C for 1 h. For negative control, an equal volume of water instead of CK1δ was added. Kinase reactions were quenched by boiling in SDS-PAGE buffer, separated by SDS-PAGE, and transferred to Immobilon-P polyvinylidene fluoride membrane (Millipore). Phosphoproteins were detected by autoradiography.

Two hundred nanograms MBP-Hhp2ΔC was added to 6 μg MBP-Arp8 WT, 3 A, or 4 A in 20 μL 1× PMP buffer supplemented with 10 mM MgCl_2_, 100 µM unlabeled ATP and 1 µCi γ-[^32^P]-ATP at 30 °C for 1 h. Reactions were quenched by boiling in SDS-PAGE buffer, separated by SDS-PAGE, stained with Coomassie, and dried. Phosphoproteins were detected by autoradiography. ^32^P was quantified on a Typhoon FLA 7000 phosphorimager.

### Mass spectrometry

TCA-precipitated MBP-Arp8 and MBP-Arp8-3A proteins from in vitro kinase assays were subjected to mass spectrometric analysis on an LTQ Velos (Thermo) by three-phase multidimensional protein identification technology (MudPIT) as previously described^91^ with the following modifications. Proteins were resuspended in 8 M urea buffer (8 M urea in 100 mM Tris pH 8.5), reduced with Tris (2-carboxyethyl) phosphine, alkylated with 2-chloro acetamide, and digested with trypsin, chymotrypsin, or elastase. The resulting peptides were desalted by C-18 spin column (Pierce). For the kinase assay samples six salt elution steps were used (i.e., 25, 50, 100, 600, 1000, and 5000 mM ammonium acetate) instead of the full 12 steps for TAP samples. Raw mass spectrometry data were first converted to MzXML files using ProteoWizard msconvert (https://proteowizard.sourceforge.io) and then converted to dta files using MzXML2Search (http://tools.proteomecenter.org/wiki/index.php?title= Software:MzXML2Search) before being searched by SEQUEST algorithm (Thermo Fisher Scientific, San Jose, CA, USA; version 27, rev. 12). Scaffold (version 4.8.4) and Scaffold PTM (version 3.2.0) (both from Proteome Software, Portland, OR) were used for data assembly and filtering. The following filtering criteria were used to analyze the phosphorylation sites: minimum of 95% peptide identification probability, minimum of 99% protein identification probability, and minimum of two unique peptides.

### Quantitative phosphoproteomics

Sample processing was performed following the SL-TMT protocol.^64^ Briefly, cell pellets were lysed in 8 M urea complemented with protease and phosphatase inhibitors by bead-beating. After lysis, the protein extracts were centrifuged and the supernatant was obtained. Samples were reduced using 5 mM TCEP for 30 min, alkylated with 10 mM iodoacetamide for 30 min, and the excess of iodoacetamide was quenched using DTT. After protein quantification, 200 µg of protein were chloroform-methanol precipitated and reconstituted in 200 µL of 200 mM EPPS (pH 8.5). Protein was digested using Lys-C overnight at room temperature followed by trypsin for 6 h at 37 °C, both at a 100:1 protein-to-protease ratio. After digestion, the samples were labeled using the TMTpro16 reagents for 60 min (Thermo), the reactions were quenched using hydroxylamine (final concentration of 0.3% v/v) for 20 min. After label check, the samples were combined equally and desalted. Phosphopeptides were enriched using the Pierce High-Select Fe-NTA Phosphopeptide Enrichment kit following manufacturer’s instructions. The phosphopeptides were eluted in a tube containing 100 µL of 10% formic acid and dried in a vacuum centrifuge. The unbound fraction was retained and fractionated using basic pH reversed-phase (BPRP) HPLC, the resulting 96 fractions were consolidated into 24, and 12 were processed in the mass spectrometer. The phosphopeptides were fractionated using the Pierce High pH Reversed-Phase Peptide Fractionation Kit following manufacturer’s instructions. The phosphopeptides were eluted using the following ACN concentrations: 7.5, 10, 12.5, 15, 17.5, 20, 22.5, 25, 27.5, 30, 40, and 50% ACN, then the samples were combined into three final fractions: (a) 7.5, 20, 22.5, and 50, (b) 10, 17.5, 25, and 40, (c) 12.5, 15, 27.5, and 30.

All data were collected on an Orbitrap Eclipse mass spectrometer coupled to a Proxeon NanoLC-1000 UHPLC. The peptides were separated using a 100 μm capillary column packed with ≈35 cm of Accucore 150 resin (2.6 μm, 150 Å; ThermoFisher Scientific). For BPRP fractions, the data were collected using a DDA-SPS-MS3 method with online real-time database searching (RTS) coupled with FAIMS.^92^^,^^93^ Each fraction was eluted using a 90 min method over a gradient from 6% to 30% ACN. Peptides were ionized with a spray voltage of 3,000 kV. The instrument method included Orbitrap MS1 scans (resolution of 120,000; mass range 400 − 1400 m/z; automatic gain control (AGC) target 2 × 10^5^, max injection time of 50 ms and ion trap MS2 scans (CID collision energy of 35%; AGC target 1 × 10^4^; rapid scan mode; max injection time of 120 ms). RTS was enabled and quantitative SPS-MS3 scans (resolution of 50,000; AGC target 2.5 × 10^5^; max injection time of 250 ms) were processed through Orbiter with a real-time false discovery rate filter implementing a modified linear discriminant analysis.

Phosphopeptides were analyzed using FAIMS/hrMS2 following our optimized workflow for multiplexed phosphorylation analysis.^92^^,^^93^ Briefly, the Thermo FAIMS Pro device was operated with default parameters. No additional gas was used for desolvation. The DV circuitry was auto-tuned, which independently tunes each of the sine waves and phase shifts one of the waveforms by π/2 to assemble a bisinusoidal waveform with a high amplitude of −5000 V at a 3 MHz frequency. The three phosphopeptide fractions were analyzed twice in the mass spectrometer, once with a method incorporating two CVs (CV = −45 and −70 V) and the other with three CVs (CV = −40 V, −60 V and −80 V) using a 2 h method using a gradient of 6% to 30% B.

Raw files were first converted to mzXML. Database searching included all *S. pombe* entries from UniProt (downloaded December 2020). The database was concatenated with one composed of all protein sequences in the reversed order and a list of common contaminant proteins was also included. Searches were performed using a 50 ppm precursor ion tolerance and 0.9 Da (low-resolution MS2) or 0.03 Da (high-resolution MS2) product ion tolerance.^94–96^ TMTpro on lysine residues and peptide N termini (+304.2071 Da for TMTpro) and carbamidomethylation of cysteine residues (+57.0215 Da) were set as static modifications (except when testing for labeling efficiency, when the TMTpro modifications are set to variable). Oxidation of methionine residues (+15.9949 Da) was set as a variable modification. For phosphopeptide analysis, +79.9663 Da was set as a variable modification on serine, threonine, and tyrosine residues. The mass spectrometry proteomics data have been deposited to the ProteomeXchange Consortium via the PRIDE^97^ partner repository with the data set identifier PXD033223.

t tests were used to compare each protein or phosphorylation site measurement, and a Benjamini − Hochberg multiple testing correction was applied. Statistical analysis and volcano plots were generated in Graph Pad Prism v8. Linear substrate motif analysis was performed on phosphopeptides with q < 0.01 using pLogo.^98^

### Statistical analysis

Statistical analysis was performed using GraphPad Prism v 8.

## Supplementary Material

Figure S2.tif

SupplementaryMaterial.docx

Figure S1_new.tif

## Data Availability

The raw data that support the findings of this study are openly available in Mendeley data at doi: 10.17632/jxkzyxn8rs.1, 10.17632/tm5hz2386j.1, 10.17632/ywmm4s53yj.1, 10.17632/86r4bzfytk.1, 10.17632/p9ghwy6sxp.1, 10.17632/mhw3x5d6mh.1, 10.17632/rgjrfvhfjc.1, and 10.17632/94yr6hrtcr.1. The mass spectrometry proteomics data have been deposited to the ProteomeXchange Consortium via the PRIDE^97^ partner repository with the data set identifier PXD033223. The authors confirm that the data supporting the findings of this study are available within the article and its supplementary materials.
