# Supplementary figures and images for: The DNA Damage Repair Function of Fission Yeast CK1 Involves Targeting Arp8, a Subunit of the INO80 Chromatin Remodeling Complex
Source: Mol Cell Biol. 2024 Oct 10;44(12):562–76. doi: 10.1080/10985549.2024.2408016 (PMC11583621; doi:10.1080/10985549.2024.2408016)

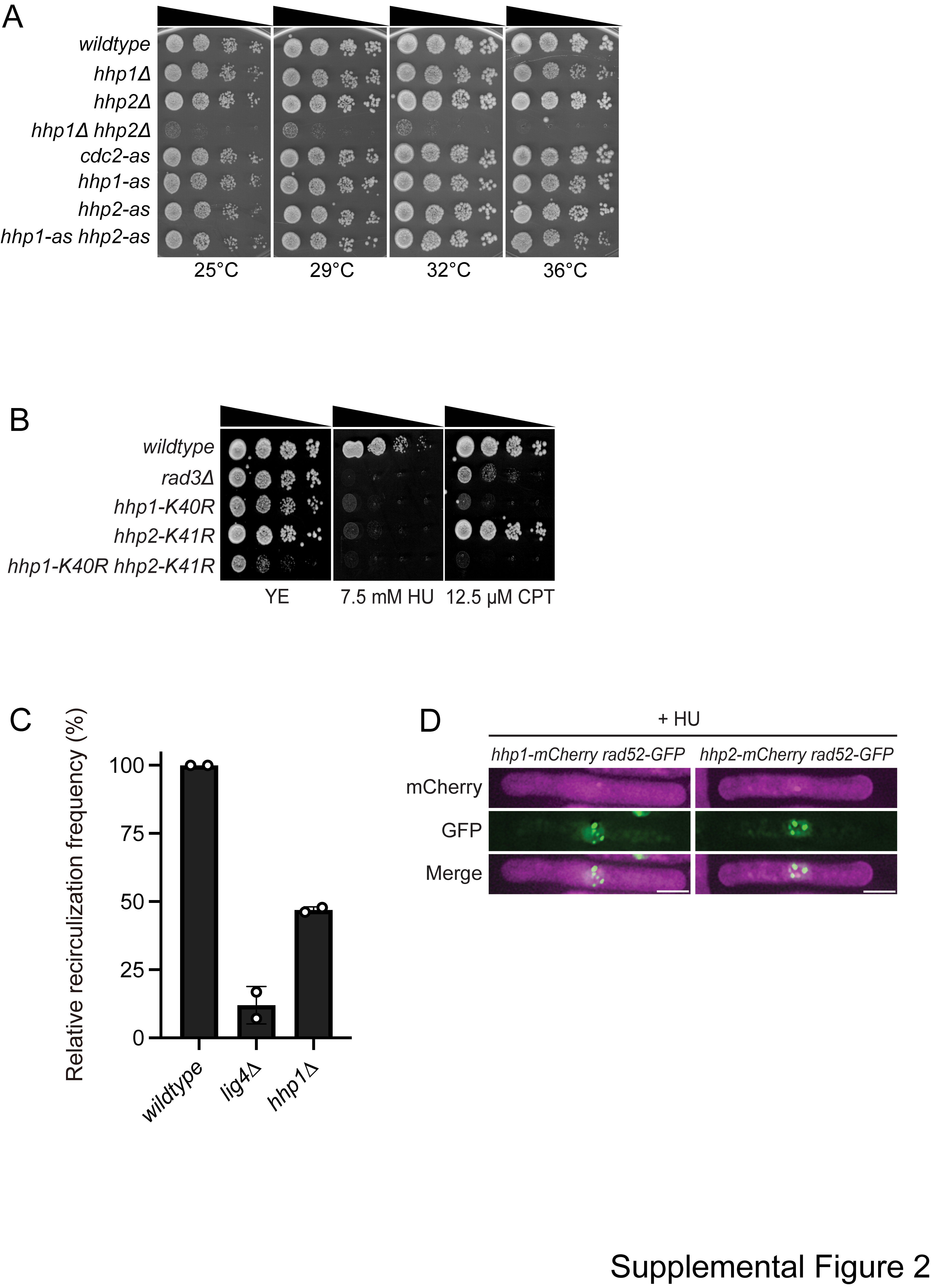

Supplement: Figure S2.tif [file TMCB_A_2408016_SM9803.tif]

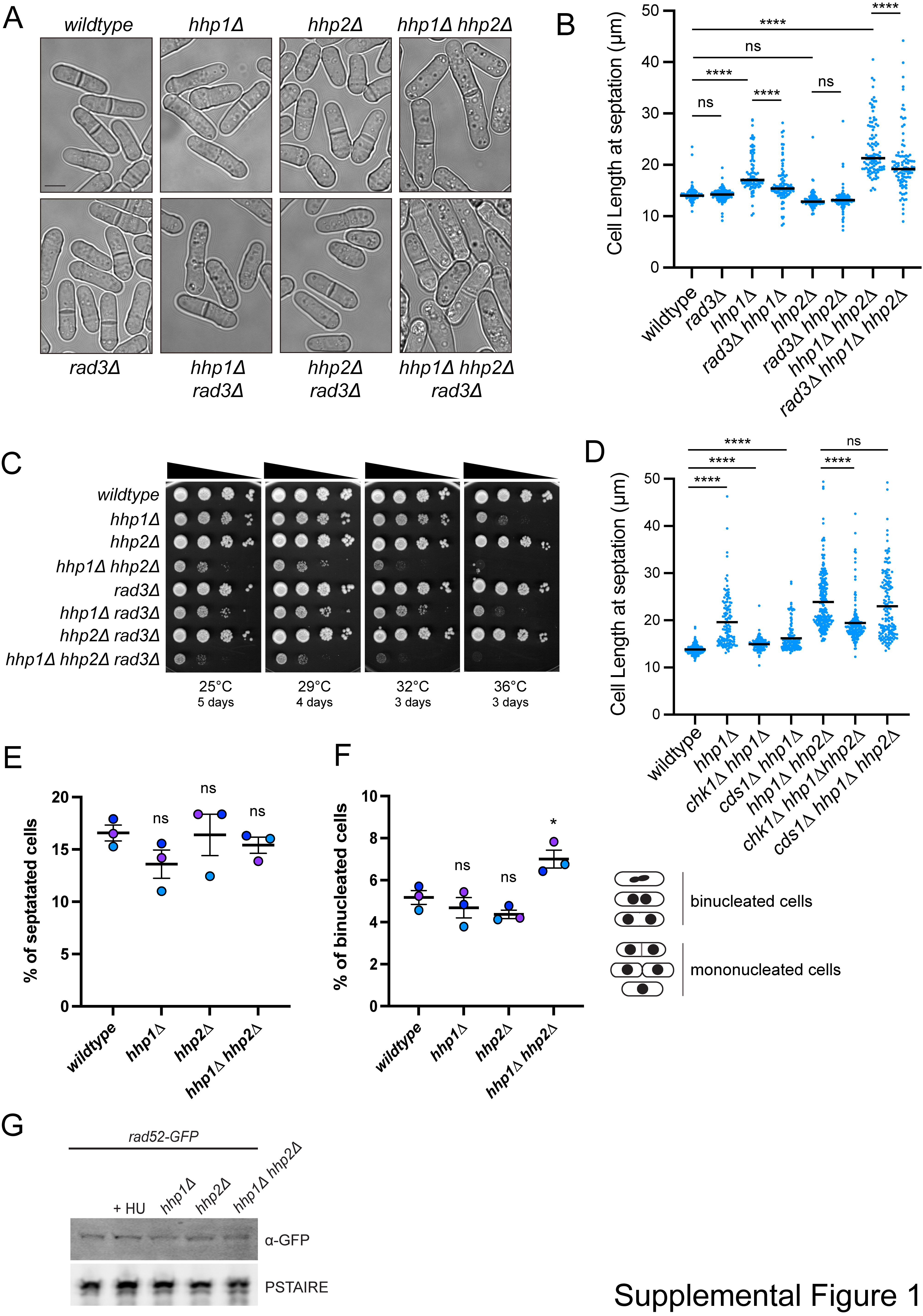

Supplement: Figure S1_new.tif [file TMCB_A_2408016_SM9801.tif]
