# Supplementary material for: The DNA Damage Repair Function of Fission Yeast CK1 Involves Targeting Arp8, a Subunit of the INO80 Chromatin Remodeling Complex
Source: Mol Cell Biol. 2024 Oct 10;44(12):562–76. doi: 10.1080/10985549.2024.2408016 (PMC11583621; doi:10.1080/10985549.2024.2408016)
Supplement: SupplementaryMaterial.docx [file TMCB_A_2408016_SM9802.docx]

**Supplementary Material**

**Figure S1: Cell cycle phenotypes of *hhp1∆, hhp2∆*, and *hhp1∆ hhp2∆* strains.** (A) DIC live-cell images of the indicated strains grown in YE at 32˚C. Scale bar: 5 μm. (B) Length at septation was quantified for cells (n ≥ 100) imaged as in A. (C) Serial 10-fold dilutions of the indicated strains were spotted on YE and incubated at the indicated temperatures. (D) Length at septation was quantified for cells (n ≥ 100 for each strain). (E,F) Quantification of septated and binucleated cells. Cells were grown in YE at 32˚C and fixed and stained with DAPI and methyl blue. n ≥ 300 in each of 3 biological replicates. Binucleated cells were counted as represented in the scheme (E). Bars represent the means. ****, p < 0.0001; *, p < 0.05; ns, not significant by one-way ANOVA. (G) Anti-GFP western blot of cell lysates from the indicated strains, with anti-Cdc2 (PSTAIRE) as the loading control.

**Figure S2: Loss of Hhp1 and Hhp2 activity confers sensitivity to DNA damaging agents.** (A,B) Serial 10-fold dilutions of the indicated strains were spotted on YE and incubated at the indicated temperatures (A) or 32˚C (B). (C) Recircularization efficiency to analyze NHEJ function. (D) Representative live cell images of the indicated strains following treatment with hydroxyurea.

**Table S1, related to Figure 4: Proteins quantified in the proteome dataset.**

**Table S2, related to Figure 4: Phosphorylation sites quantified in the phosphoproteome dataset.**

**Table S3, related to Figure 4: Gene set with HU-sensitive phenotype.**

**Table S4, *S. pombe* strains used in this study.**

Supplementary Tables can be accessed at doi: [10.17632/2kk9m94d3b.1](https://data.mendeley.com/datasets/2kk9m94d3b/1).
